# Laboratory diagnosis of the Niemann-Pick type C disease: an inherited neurodegenerative disorder of cholesterol metabolism

**DOI:** 10.1007/s11011-019-00445-w

**Published:** 2019-06-13

**Authors:** Dominika Sitarska, Agnieszka Ługowska

**Affiliations:** Department of Genetics, Institute of Psychiatry and Neurology, Al. Sobieskiego 9, 02-957 Warsaw, Poland

**Keywords:** Niemann-Pick type C disease, Oxysterols, Chitotriosidase, Bile acid metabolites, Lysosphingolipids, Filipin staining test

## Abstract

Niemann-Pick type C disease (NPC) is a genetically determined neurodegenerative metabolic disease resulting from the mutations in the *NPC1* or *NPC2* genes. It belongs to the lysosomal storage diseases and its main cause is impaired cholesterol transport in late endosomes or lysosomes. NPC is inherited in an autosomal recessive trait. Due to the wide range in age of onset, often unspecific clinical picture and varying dynamics of disease progression, the diagnosis is very difficult and long-lasting. The most characteristic visceral symptoms are hepato- or hepatosplenomegaly, which may appear independently of neurological or psychiatric symptoms at various stages of the disease. Available biochemical biomarkers should be tested as early as possible in patients presenting with hepato- or hepatosplenomegaly, long-lasting cholestatic jaundice in neonates or infantile patients, as well as in individuals at any age with: vertical supranuclear gaze palsy (VSGP), ataxia, dystonia, frontotemporal dementia and untreatable schizophrenia or psychosis. Research on biomarkers which can detect NPC patients (Cholestan-3β, 5α, 6β-triol, 7-ketocholesterol, lysosphingomyelin isoforms and bile acid metabolites) is still ongoing, although they are not specific for the NPC disease only. This mini review describes currently used diagnostic methods.

## Introduction

Niemann-Pick type C disease (NPC; OMIM #257220 and OMIM #607625) is a genetic, neurodegenerative disease classified to the group of lysosomal storage diseases, caused by the accumulation of free cholesterol and secondarily other complex lipid compounds in lysosomes or late endosomes.

This disease is caused by pathogenic mutations in *NPC1* (OMIM #607623) and *NPC2* (OMIM # 601015) genes. The *NPC1* gene encodes a large, transmembrane 142 kDa protein located in the lysosomal membrane (Bauer et al. 2002). The *NPC2* gene encodes a small (MW = 16 kDa), soluble protein with high affinity to cholesterol, located inside late endosomes or lysosomes (Ko et al. 2003). Mutations in these genes lead to disturbances in the intracellular cholesterol transport, which result in the sequestration of unesterified cholesterol and secondarily some other sphingolipids inside the lysosomes or late endosomes. In normal metabolic cycle free cholesterol liberated from lysosomes is transported to the Golgi apparatus and then esterified in the endoplasmic reticulum by Acyl-CoA cholesterol acyltransferase (ACAT) (Erickson 2013).

NPC is a rare, inherited in an autosomal recessive trait disease, whose prevalence is 1.12: 100,000 of live births for classical forms, but due to diagnostic difficulties this number is probably higher (Wassif et al. 2016).

The spectrum of the clinical picture of NPC is extremely heterogeneous. The age of onset of symptoms may vary from perinatal period to adulthood. Similarly, patients’ lifespan can range from a few days to even 60 years, but most patients die between 10 and 25 years of age (Wraith et al. 2009; Spiegel et al. 2009).

The most characteristic visceral symptoms are hepato- or hepatosplenomegaly, which may appear independently of neurological or psychiatric symptoms at various stages of the disease. Except of the small group of patients, who die at birth or in the first months of life due to liver or respiratory failures, all patients will eventually develop progressive and fatal neurological disease. Visceral symptoms, if present usually precede neurological symptoms and occur in about 85% of patients.

The most characteristic neurological symptoms are cerebellar ataxia, dysarthria, dysphagia and developmental delay or progressive dementia. The majority of patients show a characteristic vertical supranuclear gaze palsy (VSGP) (Solomon et al. 2005). Cataplexy, epileptic seizures and dystonia are also common and psychiatric symptoms, present in patients with late onset of the disease include e.g. psychosis, paranoid delusions or schizophrenia. Depending on the patient’s age at the time of the first neurological symptoms and the dynamics of disease progression several types of the disease are distinguished (Table 1) (Patterson et al. 2001; Vanier and Millat 2003; Wraith et al. 2009; Vanier 2010).

**Table 1:** Main clinical manifestations observed in different types of NPC (partly on the basis of Vanier 2010)

Type NPC	Early infantile	Late infantile	Juvenile	Adult
Systemic involvement	Hepatosplenomegaly May regress with age Absent in ~15% of cases			
Neurological involvement	- Delay in motor milestones - Hypotonia - VSGP	- Gait problems - Clumsiness - Speech delay - Cataplexy - VSGP	- School problems - Ataxia - Seizures - Cataplexy - VSGP	- Psychiatric problems - Ataxia - Dystonia - Dementia - VSGP

Early infantile type refers to patients, in whom first clinical symptoms occur between 2 months and 2 years of life. In this form of NPC hepatosplenomegaly is always present. Additionally, neurological symptoms such as psychomotor delay are observed at about 8–9 months of age and also hypotonia, which may appear between 1 and 2 years of age. Survival rarely exceeds 5 years.

In the late-infantile type of NPC first symptoms such as hepatosplenomegaly, speech delay, and walking problems caused by ataxia are observed in children between 2 and 6 years of age. VSGP, cataplexy, and epileptic seizures are common. The mental retardation progresses, dysphagia, dysarthria and dementia develop. In the later stages of the disease, pyramidal symptoms appear and spasticity and swallowing problems occur. These patients most often die between 7 and 12 years of age.

In the juvenile type of NPC the first neurological symptoms appear most often between 6 and 15 years of age and they include VSGP, difficulties in writing, disordered attention, cataplexy usually caused by laughter. Dysarthria, dysphagia, functional dystonia, severe motor delay and intellectual decline are developing. Similarly to the NPC types of earlier onset pyramidal symptoms, spasticity and swallowing problems requiring gastrostomy may appear (Vanier 2010).

In the adult type, psychiatric symptoms such as psychosis, paranoid delusions, visual and auditory hallucinations, depressive syndromes, sometimes bipolar disorder, obsessive-compulsive disorder or schizophrenia are characteristic (Kawazoe et al. 2018). The neurological symptoms may include cerebellar ataxia, dysarthria, cognitive disorders, movement disorders, and dysphagia (Sevin et al. 2007). It should be emphasized that in this type of NPC the neurological symptoms may not occur or they can be very subtle.

Although there is no a cure for NPC, the clinical symptoms of this inborn metabolic disorder can be still treated. A co-operation of a multi-disciplinary team of specialists, including neurologist, metabolist, psychiatrist, ophthalmologist, gastroenterologist, psychologist and others, brings benefits for patient’s health if introduced at an early stage of manifestations. Neurological symptoms can be attenuated by means of substrate reduction therapy (SRT) with miglustat, which is an imino sugar inhibiting the activity of glucosylceramide synthase, an enzyme involved in the biosynthesis of most glycosphingolipids. So far, this is the only approved therapy for NPC (Geberhiwot et al. 2018). Clinical trials are performed for experimental treatment with 2-hydroxypropyl-β-cyclodextrin, an activator of heat shock response (Arimoclomol) and acetyl-DL-leucine (Walterfang et al. 2012; Patterson et al. 2007, 2012; Ory et al. 2017; Bremova et al. 2015). Studies in NPC animal models demonstrated that early treatment may reduce the progress of neurological symptoms (Zervas et al. 2001).

## Laboratory diagnosis

Due to unspecific clinical signs and symptoms observed in patients affected with NPC the diagnostic process is difficult and long-lasting. Lack of one specific method giving a clear result confirming or excluding the disease demands the combination of biochemical and molecular techniques.

Available biochemical biomarkers should be tested as early as possible in patients presenting with hepato- or hepatosplenomegaly, long-lasting cholestatic jaundice in neonates or infants as well as in patients at any age with: VSGP, ataxia, dystonia, frontotemporal dementia and untreatable schizophrenia or psychosis (Patterson et al. 2017). It is also worth to remember that there is a strong suspicion of NPC in patients with unclarified hematologic problems (especially, when sea blue histiocytes are present in the bone marrow aspirates).

In this section we will shortly describe the main laboratory methods available in diagnostics of NPC (both biochemical and molecular). The filipin staining test was the first one, which has been used for identification of NPC individuals for over 30 years (Pentchev et al. 1985). Since filipin test is invasive, long lasting and laborious there was an expectation on fast and easy tests in blood. In 1994, Hollak et al. revealed chitotriosidase to be a novel biomarker for Gaucher disease but it soon was demonstrated that moderate elevation of chitotriosidase activity can be found in patients with some other lysosomal diseases and among them also in NPC (Hollak et al. 1994; Ries et al. 2006). Due to the limitation that chitotriosidase activity can remain in reference ranges, especially in NPC patients affected with late onset forms of the disorder, some novel techniques have been developed recently for biomarkers including oxysterols, lysosphingolipids, bile acid metabolites, and Bis(monoacylglycero)phosphate (BMP/LBPA). Since early 1990, molecular analyses based on polymerase chain reaction method (PCR) are available in the diagnostic process of NPC. They are particularly useful when results of biochemical biomarkers are difficult for interpretation or the filipin test is inconclusive.

### Biochemical biomarkers

#### Filipin staining test

Staining with filipin, a fluorescent chemical compound isolated from actinomycete, *Streptomyces filipinensis*, until recently was considered the gold standard in NPC diagnostics. This method is invasive because of using the skin fibroblasts taken of patients. Initially, fibroblasts are cultured in a medium deprived of cholesterol compounds to maximize the LDL receptor’s response, and then the cells are incubated for 24 h in LDL-enriched medium. Prior to staining with filipin, cells must be fixed with 10% buffered formalin (Vanier et al. 2016).

**Fig. 1:**
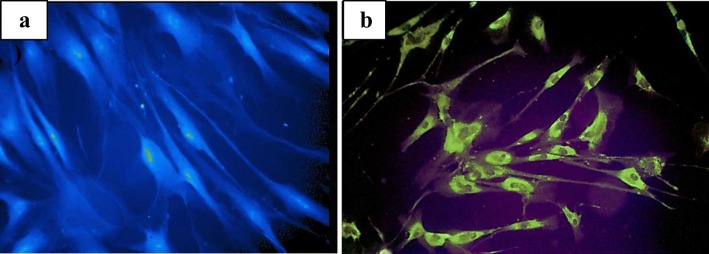
Example of filipin staining of cultured skin fibroblasts in diagnosing NPC. Cells taken from a healthy control person and patient affected with NPC were initially cultured in standard medium followed by lipoprotein deprivation for a few days and then a renewed addition of lipoprotein source to the medium. **a** Picture of control cells. **b** Multiple fluorescent granules are visible in perinuclear space (seen in fluorescent microscope). The picture shows ‘classical’ storage pattern of results seen in NPC

In fluorescence microscope, cultured NPC skin fibroblasts usually show an increased number of perinuclear luminescent vesicles, see Fig. 1. About 80–85% of cases show this ‘classical’ pattern of staining (Vanier et al. 2016). The remaining cases are referred to as the ‘variant’ biochemical phenotype, where the intensity of filipin staining is not as clear. This situation can be observed in the case of several specific mutations, e.g. p.Pro1007Ala, in some NPC heterozygotes as well as in patients with Niemann-Pick type A or B diseases (acid sphingomyelinase deficiency), lysosomal acid lipase deficiency and mucolipidosis II/III (I-cell disease) (Vanier et al. 1991).

Abnormal results of filipin staining test have also been documented in MEGDEL syndrome, Smith-Lemli-Opitz syndrome, Tangier disease, and in a rare congenital disorder of glycosylation due to Nogo-B receptor mutations (Vanier et al. 2016). Vanier et al. indicated additionally that in mouse cells deficient in mannose- 6-phosphate receptors, MPR46 and MPR300, altered filipin staining patterns have been shown (Vanier et al. 2016).

For a completed diagnosis, filipin staining should always be combined with the detection of pathogenic molecular failures in the *NPC1* and *NPC2* genes.

#### Chitotriosidase

Plasma/serum chitotriosidase activity test is most often performed for the detection of patients with Gaucher disease, in whom the elevation is significant. A moderate increase in chitotriosidase activity is also observed in individuals with NPA, NPB, NPC, lysosomal acid lipase deficiency (Wolman disease, CESD), gangliosidosis GM1, and Krabbe disease (Ries et al. 2006; Wajner et al. 2007). However, chitotriosidase activity may not always be increased in NPC patients, especially in those with late onset types of the disease. The increased activity of this enzyme is also possible in other disorders such as type II diabetes or stroke (Elmonem et al. 2016; Sotgiu et al. 2005; Bustamante et al. 2014). Noteworthy, about 10% of general population are homozygotes for chitotriosidase deficiency alleles leading to uninformative results (Ries et al. 2006).

#### Oxysterols

Oxidative stress leads to the non-enzymatic formation of cholesterol self-oxidising products and to the increased production of reactive oxygen species (ROS). In patients with NPC disease elevated levels of cholestane-3β, 5⍺, 6β-triol (C-triol) and 7-ketocholesterol (7-KC) in plasma were demonstrated (Porter et al. 2010; Jiang et al. 2011). This discovery allowed for faster diagnostics of the disease. Tests for the detection of oxysterols use gas chromatography - mass spectrometry (GC-MS) (Porter et al. 2010) or liquid chromatography - tandem mass spectrometry (LC-MS/MS) methods (Jiang et al. 2011).

However, it has been reported that in several cases of NPC patients no significant increase in the level of oxysterols or their borderline values were observed (Stampfer et al. 2013; Jiang et al. 2011; Reunert et al. 2016; Amraoui et al. 2014). Additionally, in frequent patients it is almost impossible to discriminate NPC from NPA/B individuals on the basis of only C-triol and 7-KC levels (Klinke et al. 2015; Romanello et al. 2016; Reunert et al. 2016; Pagan et al. 2015; Amraoui et al. 2014; Lin et al. 2014). The increased levels of oxysterols were also described in patients with lysosomal acid lipase deficiency (Pajares et al. 2015; Reunert et al. 2016; Pagan et al. 2015; Amraoui et al. 2014; Boenzi et al. 2014). Jiang and Romanello reported a small but significant increase in C-triol and 7-KC levels in carriers of mutation in the *NPC1* gene (in 25% and 36%, respectively) (Jiang et al. 2011; Romanello et al. 2016).

Due to their non-invasive character, low costs and short time of analysis, oxysterol tests can be used in screening for NPC. These tests are performed in small samples of serum or EDTA-plasma but it should be kept in mind, that this method requires a rigorous preanalytical procedure (Helmschrodt et al. 2014). Otherwise, false positive results are possible (especially in the case of 7-KC) due to the hemolysis or improper storage or transport of samples.

In addition to C-triol and 7-KC other oxysterols may be increased in the blood of NPC patients or animal models including: 7β-hydroxycholesterol (7β-HC), 4β-hydroxycholesterol (4β-HC), 7α-hydroxycholesterol (7α-HC) and 25-hydroxycholesterol (25-HC). In a mouse model NPC, a subset of cholesterol oxidation products (25-HC, 3β,5α,6β-triol, 7-KC, 7α-HC and 7β-HC) were all increased in the plasma of the 4 week old animals. Interestingly, in plasma samples from NPC1 patients the level of 24(S)-hydroxycholesterol (24(S)-HC) was lower than in controls (Porter et al. 2010; Ribas et al. 2016; Hammerschmidt et al. 2018).

#### Lysosphingolipids

In NPC, besides cholesterol, other lipids such as sphingolipids can be stored secondarily. Among them gangliosides are most abundant. Sphingolipids are essential components of plasma membranes in eukaryotic cells, especially in the nervous system, and they are important bioactive molecules (Malagarie-Cazenave et al. 2002). Genetic defects of enzymes or other proteins needed for their degradation lead to a subgroup of lysosomal storage diseases (LSD) called sphingolipidoses (Kolter and Sandhoff 2006).

According to their chemical structure, lysosphingolipids are deacylated derivatives of the starting sphingolipids. They are considered to be cytotoxic metabolites that contribute to the pathophysiology of LSD.

In patients with NPC, a slight increase of lysosphingomyelin (LysoSM) was demonstrated (Welford et al. 2014), and a much higher increase of its carboxylated analogue (LysoSM509) (*Giese* et al. 2015). Their levels can be examined with the use of LC-MS/MS technique. Research of Petazzoni et al. confirmed the elevation of LysoSM and LysoSM509 in the plasma of patients with NPA and NPB, and the increase of LysoSM509 without significant increase of LysoSM in patients with NPC (Pettazzoni et al. 2017). Similar results were obtained in earlier studies on these two biomarkers (Chuang et al. 2014; Welford et al. 2014; Giese et al. 2015; Polo et al. 2016; Raymond et al. 2015). Simultaneous examination of both biomarkers allows distinguishing NPA or B from NPC patients (Polo et al. 2016; Vanier et al. 2016). However, there were few cases of NPB patients described, in whom LysoSM in plasma was normal (Kuchar et al. 2017) and elevated levels of LysoSM and LysoSM509 were observed in several patients with Gaucher disease (Polo et al. 2016; Ferraz et al. 2016). Analysis of lysosphingolipids levels in plasma can be used for a screening or differential diagnosis in newborns with hepatosplenomegaly (Pettazzoni et al. 2017).

On the basis of LysoHexCer, LysoSM and LysoSM509 levels the discrimination of NPA/B, NPC, Gaucher disease, and Krabbe disease patients is possible (Pettazzoni et al. 2017; Polo et al. 2016) (please, see the Table 2).

**Table 2:** Diagnostic techniques used in recognizing NPC, overview (partly on the basis of Vanier et al. 2016)

Technique	Method	Material	Duration	Limitations
Genetic testing	Sanger Sequencing, NGS, MLPA	Low invasive - small samples of EDTA blood	Long analysis	Used as confirmatory diagnostic test because of high cost and very long analysis
Plasma oxysterol testing	LC-MS/MS or GC-MS	Low invasive - small samples of plasma (EDTA)	Rapid analysis	May be elevated in other diseases, possible false positive results due to incorrect transport or storage of samples
Lysosphingolipids testing	LC-MS/MS	Low invasive - small volume of blood	Rapid analysis	LysoSM509 does not distinguish NPC from Acid Sphingomyelinase Deficiency patients
Bile acids metabolites testing	LC-MS/MS	Very low invasive - urine, dried blood spots, plasma	Rapid analysis	May be elevated in Acid Sphingomyelinase Deficiency
Filipin staining	Cell culture, cytochemistry, fluorescence microscopy evaluation	Invasive skin biopsy	Long analysis	High cost, rigorous conditions and expertise in interpretation are critical. Some other diseases may produce positive results.

In patients with NPC and in other lysosomal storage disorders, a pathological increase in Bis(monoacylglycero)phosphate (BMP or LBPA - lysobisphosphatidic acid) was described. BMP is a structural isomer of phosphatidylglycerol, localized in the internal membranes of late endosomes where it forms specialized lipid domains. BMP is taking part especially in the control of cellular cholesterol distribution. Research of Liu and coworkers showed a 50-fold increase of di-22:6-BMP in the urine of NPC patients (Liu et al. 2014). However clinical utility of BMP in NPC diagnostics is still unknown.

#### Bile acids metabolites

Impaired cholesterol transport in patients with NPC, may result in utilization of several bile acid synthesis pathways different from these active in healthy people. In patients, a unique set of bile acids excreted in the urine has been identified. They include unsaturated C24 bile acids (5-cholenoic acids) with a sulfated 3β-hydroxyl group, a 7β-hydroxyl group, either free or conjugated with N-acetylglucosamine (GlcNAc) and the C24 carboxyl group either free or conjugated with glycine or taurine (Alvelius et al. 2001). It has been hypothesized that bile acids profile unique to NPC patients can be produced as a result of hepatic metabolism of 7-oxo-cholesterol, presumably by 3β, 7β-dihydroxy-5-cholenic acid and its 7β-N-acetylglucosamine conjugate. The results published by Maekawa showed a 400-fold increase in urinary excretion of 3β-sulfoxy-7β-N-acetyl-glucosamine-5-cholenic acid (SNAG-CA) and its conjugates with glycine (SNAG-G) and taurine (SNAG-T) (Maekawa et al. 2013). Recent studies revealed the presence of another bile acid, namely 3β, 5α, 6β-trihydroxycholanic acid and its conjugates with glycine and taurine. Jiang et al. showed that cholestane-3β, 5α, 6β-triol is a precursor for 3β, 5α, 6β-trihydroxycholanic acid, and then its transformation results in a more polar conjugate with glycine (N- (3β, 5α, 6β-trihydroxycholan-24 -oyl) glycine). The increase in the content of these acids in the plasma was 41-fold and 144-fold (respectively), which allows distinguishing NPC patients from control individuals. Another bile acid has been also identified, probably a 3β, 5α, 6β-trihydroxycholanic acid conjugate with taurine, but the increase in concentration in patients compared to control was only 6-fold higher, and in some cases its level coincided with that in healthy people (partial overlap with controls). Therefore, further research on it was discontinued (Jiang et al. 2016).

Subsequent studies on the content of 3β, 5α, 6β-trihydroxycholanic acid and N- (3β, 5α, 6β-trihydroxycholan-24-oyl) glycine in plasma in newborns and children over 1 year of age showed that the first one was present in all NPC newborn infants, while in older NPC children some results overlapped with controls. For the second of the bile acids the situation was reversed (Jiang et al. 2019). It can therefore be concluded that 3β, 5α, 6β-trihydroxycholanic acid will be an excellent marker for neonatal screening, and N- (3β, 5α, 6β-trihydroxycholan-24-oyl) glycine should be a good biomarker for the detection of children over 1 year of age affected with NPC. One limitation for these bile acids is that false positive results may occur in the case of patients with NPA and NPB, as well as with Wolman disease.

### Molecular analyses

Results of biochemical biomarkers testing alone provide a very high suspicion of NPC which in several cases must be confirmed by genetic testing. Knowledge of the molecular background of NPC is also a significant part of the genetic counseling for the affected families and can potentially serve in the prenatal diagnosing.

The most commonly used diagnostic molecular method for NPC is Sanger sequencing, which uses the polymerase chain reaction (PCR). The *NPC1* gene is located on the 18q11.2 chromosome and consists of 25 exons. Till now, 390 pathogenic mutations have been found within this gene, including 265 missense / nonsense, 28 splicing, 54 small deletions, 31 small insertions, 2 small indels, 9 large deletions and 1 large insertion / duplication. The *NPC2* gene located on the 14q24.3 chromosome consists of 5 exons. Till now, 23 mutations within this gene have been described, 17 missense / nonsense, 3 splicing and 3 small deletions (http://www.hgmd.cf.ac.uk/ 18.01.2019). In 95% of NPC cases, pathogenic mutations are located in the *NPC1* gene, and only 5% in the *NPC2* gene (Patterson et al. 2012; Vanier et al. 2016). Recently, Polese-Bonatto et al. defined frequent mutations in *NPC1* gene and revealed 5 novel mutations in Brazilian NPC patients (Polese-Bonatto et al. 2019). The most frequent mutation in this cohort was p.Ala1035Val (27.0%), followed by p.Pro1007Ala (16.9%), and p.Phe1221Serfs*20 (14.6%). Novel *NPC1* variants were: one small deletion (p.Lys38_Tyr40del), one frameshift (p.Asn195Lysfs*2), and 3 missense mutations (p.Cys238Arg, p.Ser365Pro, and p.Val694Met).

The rare causes of NPC disease may also be deletions of exons or whole genes detected by MLPA (multiplex ligation-dependent probe amplification) as well as mutations in the promoter region of the gene and deeply intronic. These changes can only be detected by next-generation sequencing (NGS)) (Vanier et al. 2016).

All biochemical and molecular techniques described in this review are summarized in Table 2.

## Conclusions

This review summarizes currently used diagnostic methods in recognizing NPC disease. Oxysterols, lysosphingomyelin isoforms and bile acid metabolites appear to be rapid, low-invasive and inexpensive methods for initial screening, although they display certain limitations. These biochemical biomarkers may be increased also in other lysosomal diseases leading to false positive diagnoses. And on the contrary, results within the reference range do not necessarily exclude NPC. Biochemical biomarkers do not deliver a definite diagnosis and must be confirmed by molecular analyses such as Sanger sequencing or NGS. If a mutation of unknown pathogenicity is detected, the filipin staining test must also be performed in cultured skin fibroblasts.
